# Content Validation of Primary Care Network Assessment Scale for Palliative Care Patients (ARCP) and Clinical Utility in Patients With Advanced Cancer

**DOI:** 10.1111/scs.70303

**Published:** 2026-08-06

**Authors:** Érika Fernanda Palmieri Guimarães Fontes, Andréia Pereira Assis de Ouverney, Karla Santos da Costa da Rosa, Lívia Costa de Oliveira

**Affiliations:** ^1^ Palliative Care Unit National Cancer Institute (INCA) Rio de Janeiro Brazil

**Keywords:** caregivers, oncology, palliative care, primary care network, quality of life, social support

## Abstract

**Objective:**

To validate the content of Primary Care Network Assessment Scale for Palliative Care Patients (ARCP) and to assess its clinical utility in patients with advanced cancer.

**Methods:**

This methodological study was conducted in two phases. Phase 1 involved content validation by ten social work experts using the Content Validity Index (CVI). Phase 2 evaluated clinical utility in a cross‐sectional study of 148 patients with advanced cancer receiving palliative care. Data included sociodemographic and clinical variables, global quality of life (EORTC QLQ‐C15‐PAL) and perceived social support (SSQ6). Multivariate logistic regression analyses were performed.

**Results:**

The ARCP showed high content validity, with CVI values above 0.80 for relevance, clarity and vocabulary across all items. Clinically, 80% of patients were classified as having fully participatory caregiving networks; however, 42% relied on a single caregiver, indicating structural vulnerability despite high perceived support. Male sex (OR = 2.53; 95% CI: 1.02–4.34), being married (OR = 2.78; 95% CI: 1.06–7.29), better global quality of life (OR = 2.33; 95% CI: 1.08–2.57) and greater satisfaction with social support (OR = 5.41; 95% CI: 1.35–15.05) were independently associated with a fully participatory network.

**Conclusion:**

The ARCP demonstrated evidence of content validity and clinical utility for assessing caregiving network structure in palliative care.

## 1 Introduction

Palliative care represents a proactive and comprehensive approach that shifts the focus from disease treatment to the patient–family dyad, recognizing that psychological adjustment, emotional suffering and quality of life are deeply influenced by the availability and organization of social and caregiving relationships. This model demands rigorous multidisciplinary action to manage the physical, psychological and social dimensions of the care plan, with a particular emphasis on the structure and dynamics of the support network [1]. Care networks can be categorized as primary or secondary, distinguished by the nature of the exchanges and bonds established [2]. Primary networks, predominantly anchored in familial and affective ties, such as friends and neighbours, are characterized by bonds of trust and reciprocity [3]. Understanding the composition, proximity and caregiving capacity of these networks is fundamental to designing a person‐centered care plan tailored to individual needs [4].

However, the support needs of family caregivers are frequently underestimated in clinical practice, despite consistent evidence that caregiving burden, emotional distress and lack of preparedness significantly affect both caregiver well‐being and patient outcomes in advanced illness. Recent evidence shows that caregivers of individuals with serious illness often experience a mismatch between expected and received support, highlighting the need to assess not only perceived support but also the structural and relational dimensions of caregiving networks [5]. Studies demonstrate that the use of assessment instruments, such as the Carer Support Needs Assessment Tool (CSNAT), can improve communication and reduce burden in palliative settings [6]. Nevertheless, even well‐validated measures of social support have focused primarily on perception and functional aspects, rather than on the structural configuration and sustainability of care networks [7].

Scoping reviews of caregiver interventions also highlight that measurement tools rarely capture the relational dynamics of caregiving networks, which may limit the identification of psychosocial risk factors for both caregivers and patients [8]. In Brazil, and to a large extent internationally, the development of tools specifically designed to evaluate the structural configuration and functional capacity of caregiving networks in palliative care remains limited. To address this gap, the Brazilian National Cancer Institute (INCA) developed the Primary Care Network Assessment Scale for Palliative Care Patients (ARCP) in 2016, and was later incorporated into institutional palliative care practice. Although routinely used in clinical assessments, no formal content validation or evaluation of its clinical utility had been conducted.

This tool, based on an integrative literature review and the systematization of professional social work practice [9, 10, 11, 12, 13, 14, 15], enables multidisciplinary teams to classify the patient's care network based on affective ties, the number of involved caregivers, and the comprehensiveness of care provided. Rather than measuring a single latent construct, the ARCP was designed as a hierarchical clinical classification framework that integrates four dimensions of caregiving organization: affective bonds, caregiver participation, number of caregivers and comprehensiveness of care provision. The ARCP offers a practical response to aspects often unmeasured in current literature [16].

Given the lack of instruments to evaluate the structural configuration of support networks, this study aimed to validate the content of ARCP and to assess its clinical utility in patients with advanced cancer.

## 2 Methods

### 2.1 Study Design and Ethical Considerations

This methodological, cross‐sectional study was conducted at the Palliative Care Unit of the INCA. The research was divided into two distinct phases: (1) content validation of the ARCP and (2) an assessment of its clinical utility. The study was approved by the Institutional Research Ethics Committee (CAAE: 60656922.8.0000.5274), and all participants provided written informed consent.

### 2.2 Phase 1: Content Validation

The content validation was conducted using a modified Delphi‐like approach to ensure the scale's relevance, clarity, and vocabulary appropriateness. To compose the expert panel, 14 social workers with recognized expertise in palliative care were intentionally invited to participate. Selection criteria included: (a) a minimum of 5 years of clinical experience in oncology or palliative care; (b) professional affiliation with reference institutions; and (c) specialized postgraduate training in the field. This sample size was designed to ensure a diverse range of professional perspectives and to maintain the stability of the consensus, as recommended in methodological literature for instrument validation [17, 18, 19].

The invited experts received a digital form containing the scale's items and a structured evaluation matrix. Each item was assessed using a 5‐point Likert scale (ranging from 1‐“Not relevant/clear” to 5‐“Highly relevant/clear”). Consensus was predefined as a Content Validity Index (CVI) of ≥ 0.80 for each evaluated criterion. If an item failed to meet this threshold, it would be revised and resubmitted for additional rounds of review until an acceptable level of agreement was achieved [17, 18, 19].

### 2.3 Phase 2: Clinical Utility

To evaluate the clinical utility of the ARCP, we conducted an observational, cross‐sectional study involving patients with advanced cancer receiving care at the Palliative Care Unit of the INCA.

#### 2.3.1 Eligibility Criteria

Eligible participants were adults aged over 20 years with a confirmed diagnosis of advanced cancer who were under follow‐up at the INCA Palliative Care Unit. Additional inclusion criteria included a Karnofsky Performance Status (KPS) ≥ 30% [20], ensuring the ability to participate in clinical interviews. Patients presenting with cognitive impairment or clinical instability that could compromise reliable self‐reporting were excluded from the study.

#### 2.3.2 Sample Size Calculation

The minimum sample size was estimated based on the “one in ten” rule for logistic regression, which recommends at least ten outcome events per explanatory variable to ensure model stability and minimize overfitting [21]. Considering a final multivariate model with approximately six independent variables and assuming a conservative outcome prevalence of 50%, a minimum sample of 120 participants was required. This approach was adopted to ensure statistical robustness while acknowledging the exploratory nature of the clinical utility analysis.

#### 2.3.3 Data Collection and Instruments

Data were collected by a trained researcher through structured clinical interviews and a comprehensive review of electronic medical records. The following instruments and variables were utilized:

### 2.4 ARCP

This a multidisciplinary, professional‐rated assessment tool designed to evaluate the structural configuration and functional capacity of caregiving networks in palliative care. In this study, all assessments were performed by a social worker trained and experienced in palliative care, based on information obtained from patients and, whenever available, the primary caregiver. The scale classifies caregiving networks into eight hierarchical categories, ranging from an absent network to networks characterized by varying levels of caregiver participation, number of caregivers involved and comprehensiveness of care provision. Classification is based on four core dimensions: (a) presence of family or affective bonds; (b) presence and degree of caregiver participation; (c) number of caregivers; and (d) quality and comprehensiveness of care provided.

For analytical purposes, ARCP categories 1 to 5 were grouped as non‐participatory or partially participatory networks, whereas categories 6 to 8 were classified as fully participatory networks. This dichotomization was adopted to enable binary logistic regression analyses and was guided by the conceptual distinction between networks providing only partial caregiving coverage (categories 1–5) and those providing comprehensive caregiving coverage (categories 6–8). Although this approach does not capture differences in network size and composition across individual categories, it allowed the identification of factors associated with the availability of comprehensive caregiving support.

Global quality of life over the past week: Assessed using item 15 of the EORTC QLQ‐C15‐PAL, with responses rated on a 7‐point scale (1‐“very poor” to 7‐“excellent”) [22].

Perceived Social Support: The short version of the Social Support Questionnaire (SSQ6) evaluated the number of available support persons and the patient's satisfaction with said support [23].

Sociodemographic and clinical variables: Data included age, sex, self‐reported skin colour, education level, marital status, religion and per capita income. Clinical variables included primary tumour site, metastasis, prior treatment, comorbidities and KPS [20].

#### 2.4.1 Statistical Analysis

Statistical analyses were performed using Stata Data Analysis and Statistical Software, version 15.0 (StataCorp, College Station, TX, USA). Categorical variables were summarized using absolute and relative frequencies, and group comparisons were conducted using the chi‐square test or Fisher's exact test, as appropriate. A *p*‐value < 0.05 was considered statistically significant.

Binary logistic regression was employed to identify factors associated with the presence of a fully participatory caregiving network. Variables with *p*‐values < 0.20 in univariate analyses or with established theoretical relevance to caregiving dynamics and psychosocial vulnerability were included in the multivariate model. A backward stepwise selection procedure was applied, with variables sequentially removed based on descending *p*‐values, and only those with *p* < 0.05 retained in the final adjusted model. Results are presented as odds ratios (OR) with corresponding 95% confidence intervals (CI).

## 3 Results

### 3.1 Phase 1: Content Validation

Of the 14 social workers invited to participate in the content validation process, 12 responded (response rate: 86%). Two responses were excluded due to inconsistencies, resulting in a final expert panel of 10 participants. The panel had a mean of 12 years of professional experience in oncology and palliative care. Most experts were from the Southeast (61%) and Northeast (23%) regions of Brazil and predominantly worked in public healthcare services (58%) (data not shown).

In a single round of the modified Delphi process, the ARCP demonstrated high levels of expert agreement. The CVI exceeded the predefined threshold of 0.80 for relevance, clarity and vocabulary appropriateness across all items, with global CVI values ranging from 0.86 to 0.92 (Table 1). Qualitative feedback was limited and primarily related to minor semantic adjustments to enhance cultural clarity. After review by the research team, one suggestion per item was incorporated, resulting in the final version of the ARCP (Tables 2 and S1).

**TABLE 1:** Content validation of the Primary Care Network Assessment Scale for palliative care patients (*Escala de Avaliação da Rede Primária de Cuidados para Pacientes em Cuidados Paliativos—*ARCP).

Item	Types of primary care network	CVI		
		Relevance	Clarity	Vocabulary appropriateness
1	Absent	0.90	0.80	0.80
2	Present/Non‐participatory^a^	1.00	0.80	0.80
3	Present/Participatory/Restricted/Partial	0.90	0.90	0.90
4	Present/Participatory/Moderate/Partial	0.80	0.80	0.80
5	Present/Participatory/Broad/Partial	0.80	0.80	0.80
6	Present/Participatory/Restricted/Fully	1.00	0.90	0.90
7	Present/Participatory/Moderate/Fully	0.90	0.90	0.90
8	Present/Participatory/Broad/Fully	0.90	0.90	0.90
	Global CVI	0.90	0.85	0.85

Abbreviation: CVI = Content Validity Index.

^a^ This refers to a social network that exists but does not engage in caregiving.

**TABLE 2:** English final version of the Primary Care Network Assessment Scale for patients in palliative care (ARCP).

Classification	Bond type	Presence of family or emotional bonds	Presence of caregiver	Number of caregivers	Quality of care	Description
1	Social network	Absent	Not applicable	Not applicable	Not applicable	No family or emotional ties identified to provide care for the patient.
2	Social network	Present	Non‐participatory	Not applicable	Not applicable	Family and/or emotionally close individuals are identified, but they do not organize themselves as a care network.
3	Primary care network	Present	Participatory	Restricted (1 caregiver)	Partial	A single caregiver is identified, but they do not meet all of the patient's care needs.
4	Primary care network	Present	Participatory	Moderate (2 caregivers)	Partial	Two caregivers are involved, but their actions do not meet all of the patient's care needs.
5	Primary care network	Present	Participatory	Broad (3+ caregivers)	Partial	Three or more caregivers are involved, but they do not fully meet all of the patient's care needs.
6	Primary care network	Present	Participatory	Restricted (1 caregiver)	Full	A single caregiver is identified who fully meets all of the patient's care needs.
7	Primary care network	Present	Participatory	Moderate (2 caregivers)	Full	Two caregivers are involved and together fully meet the patient's care needs.
8	Primary care network	Present	Participatory	Broad (3+ caregivers)	Full	Three or more caregivers are involved and together fully meet the patient's care needs.

### 3.2 Phase 2: Clinical Utility

**FIGURE 1:**
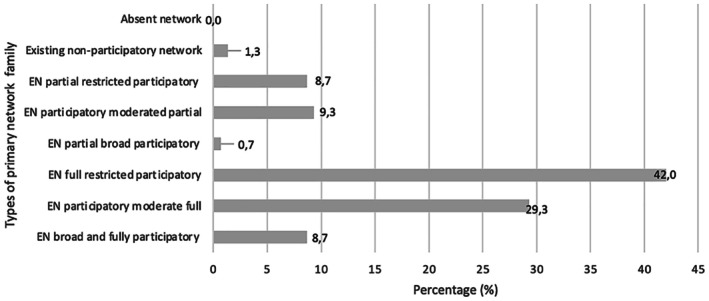
Primary care network of patients with advanced cancer receiving palliative care, according to the ARCP (*n* = 148). ARCP = Primary Care Network Assessment Scale for Palliative Care Patients; EN = existing network.

A total of 148 patients with advanced cancer receiving palliative care were included in the clinical utility phase. Although most patients (80%) were classified as having fully participatory caregiving networks according to the ARCP, a substantial proportion (42%) relied on a single caregiver, indicating structural vulnerability within otherwise participatory networks. Only 8.7% of participants had a broad network consisting of three or more caregivers (Figure 1).

The majority of participants were older than 60 years (63.5%) and female (52.0%). A higher proportion of men were classified as having a fully participatory caregiving network compared with women (87.3% vs. 72.7%, *p* = 0.027). Similarly, married individuals were more likely to have a fully participatory caregiving network compared to those who were single, divorced, or widowed (*p* = 0.017) (Table 3). No significant differences were observed according to age group, skin colour, education level, religion, or per capita income.

**TABLE 3:** Sociodemographic profile of patients with advanced cancer receiving palliative care, according to the classification of the care network using the ARCP (*n* = 148).

Variables	Total N (%)	Non‐participatory or partially participatory network (*n* = 30; 20.0%)	Fully participatory network (*n* = 118; 80.0%)	*p*‐value*
Age (years)				
< 60	54 (35.5%)	11 (20.4%)	43 (79.6%)	0.982
≥ 60	94 (63.5%)	19 (20.2%)	75 (79.8%)	
Sex				
Male	71 (48.0%)	9 (12.7%)	62 (87.3%)	**0.027**
Female	77 (52.0%)	21 (27.3%)	56 (72.7%)	
Skin colour				
White	61 (41.2%)	14 (22.9%)	47 (77.1%)	0.497
Black and Brown	87 (58.8%)	16 (18.4%)	71 (81.6%)	
Education level				
Up to elementary school	92 (62.2%)	17 (18.5%)	75 (81.5%)	0.487
High school or above	56 (37.8%)	13 (23.2%)	43 (76.8%)	
Marital status				
Single/Divorced/Widowed	85 (57.5%)	23 (27.1%)	62 (72.9%)	**0.017**
Married	63 (42.5%)	7 (11.1%)	56 (88.9%)	
Catholic religion				
Yes	67 (45.3%)	9 (13.3%)	58 (86.7%)	0.060
No^a^	81 (54.7%)	21 (25.9%)	60 (74.1%)	
*Per capita* income (> 940 R$)^b^				
Yes	72 (48.6%)	16 (22.2%)	56 (77.8%)	0.565
No	76 (51.4%)	14 (18.4%)	62 (81.6%)	

*Note:* ARCP = Primary Care Network Assessment Scale for Palliative Care Patients; TFD = Out‐of‐Town Treatment Program. Bold values are statistically significant (*p*‐value < 0.050).

^a^ Includes Evangelical, Umbanda, Kardecist and no religion.

^b^ R$940 = median per capita income.

* Chi‐square test for proportions or Fisher's exact test.

Regarding clinical characteristics, head and neck cancer was the most prevalent tumour site (21.6%). Most patients had metastatic disease (89.2%), a KPS above 50% (60.1%) and reported good to excellent global quality of life (66.2%). Patients reporting better global quality of life were more frequently classified as having a fully participatory caregiving network than those reporting poorer quality of life (84.7% vs. 70.0%, *p* = 0.035). Similarly, the proportion of participants classified as having a fully participatory caregiving network was higher among those satisfied with their social support compared with those who were dissatisfied or neutral (82.0% vs. 44.4%, *p* = 0.007) (Table 4). No significant associations were observed between network classification and tumour site, presence of metastases, comorbidities, prior oncological treatment, or functional status.

**TABLE 4:** Clinical profile, quality of life and social support of patients with advanced cancer receiving palliative care, according to the classification of the care network using the ARCP (*n* = 148).

Variables	Total *N* (%)	Non‐participatory or partially participatory network (*n* = 30; 20.0%)	Fully participatory network (*n* = 118; 80.0%)	*p*‐value*
Primary tumour site				
GIT^a^	31 (20.9%)	5 (16.1%)	26 (83.9%)	0.489
Gynaecological^b^	30 (20.3%)	7 (23.3%)	23 (76.7%)	
HNC	32 (21.6%)	7 (21.9%)	25 (78.1%)	
Breast	14 (9.5%)	5 (35.7%)	9 (64.3%)	
Others^c^	41 (27.7%)	6 (14.6%)	35 (85.4%)	
Metastasis				
Local only	16 (10.8%)	2 (12.5%)	17 (87.5%)	0.413
Local and distance	132 (89.2%)	28 (21.2%)	104 (78.8%)	
DM				
No	139 (93.9%)	28 (20.1%)	111 (79.9%)	0.881
Yes	9 (6.1%)	2 (22.2%)	7 (77.8%)	
SAH				
No	109 (73.7%)	24 (22.0%)	85 (78.0%)	0.376
Yes	39 (26.3%)	6 (15.4%)	33 (84.6%)	
Previous treatment				
No	26 (17.6%)	3 (11.5%)	23 (88.5%)	0.223
Yes	122 (82.4%)	27 (22.1%)	95 (77.9%)	
KPS (%)				
≤ 50	59 (39.9%)	10 (16.9%)	49 (83.1%)	0.413
> 50	89 (60.1%)	20 (22.5%)	69 (77.5%)	
Overall quality of life				
Very bad to neutral	50 (33.8%)	15 (30.0%)	35 (70.0%)	**0.035**
Good to great	98 (66.2%)	15 (15.3%)	83 (84.7%)	
Satisfaction with social support				
Dissatisfied to neutral	9 (6.1%)	5 (55.6%)	4 (44.4%)	**0.007**
Satisfied	139 (93.9%)	25 (18.0%)	114 (82.0%)	

*Note:* ARCP = Primary Care Network Assessment Scale for Palliative Care Patients; GIT = gastrointestinal tract; HNC = head and neck cancer; SAH = systemic arterial hypertension; DM = diabetes mellitus; KPS = Karnofsky Performance Status. Bold values are statistically significant (*p*‐value < 0.050).

^a^ Oesophagus, stomach, intestine and rectum.

^b^ Cervix and ovary.

^c^ Connective tissue, lung, central nervous system, skin, thyroid, kidney and prostate.

* The *p*‐value refers to the Chi‐square test for proportions or Fisher's exact test.

In univariate logistic regression analyses, male sex, being married, better global quality of life and higher satisfaction with social support were associated with the presence of a fully participatory caregiving network (Table 5). These variables were retained in the multivariate model. After adjustment, male patients had higher odds of being classified as having a fully participatory caregiving network compared to female patients (OR = 2.53; 95% CI: 1.02–4.34). Being married was also independently associated with a fully participatory caregiving network (OR = 2.78; 95% CI: 1.06–7.29).

**TABLE 5:** Factors associated with a fully participatory primary care network among patients with advanced cancer receiving palliative care, according to the ARCP (*n* = 148).

Variables	Univariate			Multivariate^a^		
	OR	95% CI	*p*‐value	OR	95% CI	*p*‐value
Age (years)						
< 60	1					
≥ 60	1.01	(0.44–2.32)	0.982			
Sex						
Male	2.58	(1.09–6.10)	**0.031**	2.53	(1.02–4.34)	**0.046**
Female	1			1		
Skin colour						
White	1					
Black and Brown	0.76	(0.38–1.69)	0.498			
Education level						
Up to elementary school	1					
High school or above	1.33	(0.59–3.01)	0.488			
Marital status						
Single/Divorced/Widowed	1			1		
Married	2.97	(1.18–7.45)	**0.020**	2.78	(1.06–7.29)	**0.038**
Catholic religion						
Yes	1.25	(0.95–5.33)	0.070			
No	1					
*Per capta* income (> 940 R$)						
Yes	1					
No	1.07	(0.59–1.93)	0.566			
Primary tumour site						
GIT	1					
Gynaecological	1.63	(0.65–4.12)	0.299			
HN	1.01	(0.42–2.44)	0.979			
Breast	0.95	(0.28–3.22)	0.935			
Outhers	1.22	(0.54–2.76)	0.624			
Metastasis						
Local only	1.88	(0.74–4.56)	0.420			
Local and distance	1	(0.41–8.86)				
DM						
No	1					
Yes	2.12	(0.60–7.53)	0.881			
SAH						
No	1					
Yes	1.36	(0.70–2.65)	0.379			
Previous treatment						
No	1.85	(0.86–3.97)	0.232			
Yes	1					
KPS (%)						
≤ 50	1.42	(0.61–3,30)	0.415			
> 50	1					
Overall quality of life						
Very bad to neutral	1			1		
Good to great	2.37	(1.05–2.97)	**0.038**	2.33	(1.08–2.57)	**0.045**
Satisfaction with social support						
Dissatisfied to neutral	1			1		
Satisfied	5.70	(1.43–17.78)	**0.014**	5.41	(1.35–15.05)	**0.018**

*Note:* ARCP = Primary Care Network Assessment for Patients in Palliative Care; OTP = Out‐of‐Home Treatment Program; GIT = gastrointestinal tract; HN = head and neck; DM = diabetes mellitus; SAH = systemic arterial hypertension; KPS = Karnofsky Performance Status; OR = odds ratio; 95% CI = 95% confidence interval. Bold values are statistically significant (*p*‐value < 0.050).

^a^ All variables with a *p*‐value < 0.200 in the univariate analysis were entered into the multiple model simultaneously. Then, variables were removed one by one in decreasing order of ^1^ *p*‐value. Variables with a *p*‐value < 0.05 were retained in the model.

Among psychosocial variables, satisfaction with social support emerged as the factor most strongly associated with a fully participatory caregiving network (OR = 5.41; 95% CI: 1.35–15.05). In addition, patients reporting better global quality of life had higher odds of being classified as having a fully participatory caregiving network (OR = 2.33; 95% CI: 1.08–2.57).

## 4 Discussion

The content validation and clinical assessment of the ARCP represent an important step toward a more granular understanding of caregiving networks in palliative care from a psychosocial and relational perspective. While many tools emphasize patients' or caregivers' subjective perceptions of support and needs [24, 25, 26, 27], the ARCP adds value by providing a professional‐rated characterization of the network's structural configuration and functional caregiving capacity, which may reveal vulnerabilities that remain “invisible” when only satisfaction or perceived support are measured.

A central finding of this study is the coexistence of high perceived satisfaction with social support and structurally fragile caregiving arrangements, evidenced by the substantial proportion of single‐caregiver configurations. This paradox suggests that perceived support may not fully capture the sustainability of caregiving structures in advanced illness. In psycho‐oncology, this mismatch is clinically relevant because hidden relational vulnerabilities may delay timely psychosocial intervention and contribute to caregiver strain. Recent dyadic research in advanced cancer supports the interdependence between patient–caregiver burden, psychological distress and quality of life, reinforcing that caregiving structure can shape psychosocial outcomes at the dyad level [28].

The high frequency of restricted networks highlights the psychosocial risks of single‐caregiver arrangements in the context of progressive, multidimensional demands. Even when emotional bonds are strong and patients report being satisfied with support, concentrating caregiving responsibilities in one individual increases the likelihood of caregiver overload, emotional exhaustion and potential burnout [29, 30]. Contemporary evidence in palliative care has emphasized the need to identify caregiver burden early and to implement targeted support strategies and dyad‐focused interventions that strengthen coping, communication and psychological well‐being [31, 32, 33].

Notably, a considerable proportion of fully participatory networks relied on a single caregiver. This finding illustrates that comprehensive caregiving coverage and structural vulnerability are not mutually exclusive. A caregiving network may be classified as fully participatory because identified care needs are met, while still remaining vulnerable due to its reliance on a single caregiver. This distinction reinforces the importance of examining individual ARCP categories in addition to the dichotomized classification used for regression analyses. The dichotomized classification was adopted primarily for analytical purposes and should not be interpreted as superseding the clinical and conceptual relevance of the full ARCP classification framework.

In this study, higher satisfaction with social support and better global quality of life were independently associated with fully participatory networks. These findings are consistent with recent evidence showing that psychosocial and relational resources, particularly social support and social functioning, are associated with better quality of life and lower distress across the cancer trajectory, including in palliative care contexts [34]. Moreover, dyadic and caregiver‐focused studies suggest that social support is intertwined with caregiver burden and quality of life, supporting its relevance as a protective psychosocial factor in advanced illness [35, 36, 37]. Importantly, the ARCP complements perception‐based measures by operationalizing caregiving capacity and network sustainability, helping clinicians identify psychosocial risk even when reported satisfaction with support is high [5].

Gender and marital status were independently associated with the presence of a fully participatory caregiving network, revealing sociocultural patterns that are highly relevant to psycho‐oncology. Women are often socially positioned as “default caregivers”; however, when they become patients, they may have less access to diversified and sustainable caregiving networks, potentially reflecting gendered inequities in relational support. Likewise, the higher prevalence of fully participatory caregiving networks among married participants highlights the central role that spouses often assume in caregiving arrangements. These findings suggest that ARCP can contribute to identifying relational inequities and to tailoring psychosocial care planning [38, 39].

These findings underscore the importance of considering caregiving networks as dynamic relational systems that shape psychosocial vulnerability and protective resources in advanced cancer. By operationalizing caregiving structure and capacity, the ARCP contributes to a more nuanced understanding of relational risk within psycho‐oncology and palliative care.

### 4.1 Clinical and Research Implications

Our findings underscore the clinical relevance of assessing caregiving networks beyond subjective perceptions of social support. Structurally fragile networks, particularly those relying on a single caregiver, may function as markers of relational psychosocial vulnerability, informing proactive multidisciplinary care planning and more strategic allocation of psychosocial and supportive resources to prevent care‐related crises and caregiver overload.

Clinically, the ARCP can be integrated into routine palliative care assessments as a relational psychosocial triage tool, complementing perception‐based measures of social support and quality of life. By providing a professional‐rated evaluation of caregiving structure and capacity, the ARCP enables early identification of patients and families who may benefit from targeted interventions, including caregiver education, redistribution of caregiving responsibilities, engagement of extended family or community resources and timely referral to psychosocial support services.

From a research perspective, the ARCP offers a standardized approach to operationalizing caregiving network structure, supporting future longitudinal and multicenter studies examining associations between network configurations and psychosocial outcomes, caregiver burden trajectories and healthcare utilization. Further research is warranted to evaluate whether ARCP‐guided interventions can improve patient and caregiver quality of life and reduce psychosocial distress across the course of advanced cancer.

### 4.2 Study Limitations and Scientific Caution

This study has notable strengths. First, it advances the field by validating a tool that captures the structural and functional dimensions of caregiving networks through professional‐rated assessment, complementing perception‐based measures. Second, the two‐phase design (content validation and clinical utility) supports methodological rigour and practical applicability in real‐world palliative care.

Limitations should also be acknowledged. The single‐center design at a national reference institute may limit generalizability to other regions and healthcare contexts. In addition, the cross‐sectional analysis precludes causal inferences and does not capture changes in network configuration over time. Further psychometric work is warranted, including reliability (e.g., interrater and test–retest), responsiveness to change and predictive validity in longitudinal studies. Because the ARCP was conceived as a hierarchical clinical classification framework rather than a traditional psychometric scale measuring a single latent construct, future studies should also explore the performance and interpretability of its individual categories across different palliative care settings. Finally, although our findings align with the growing literature on caregiver burden and dyadic psychosocial processes in advanced cancer, further studies should investigate whether ARCP‐guided interventions can reduce caregiver strain and improve patient–caregiver outcomes.

## 5 Conclusion

The ARCP demonstrated evidence of content validity and clinical utility for assessing caregiving network structure in palliative care. By moving beyond subjective perceptions of support, it helps identify relational psychosocial vulnerabilitie**s** that may place patients and caregivers at risk, supporting earlier and more targeted multidisciplinary and psychosocial interventions in advanced cancer care.

## Author Contributions

All authors contributed to the study conception and design. Material preparation, data collection, and analysis were performed by É.F.P.G.F., A.P.A.O., K.S.C.R., and L.C.O.; The first draft of the manuscript was written by É.F.P.G.F., and all authors commented on subsequent versions of the manuscript. All authors read and approved the final manuscript.

## Funding

The authors have nothing to report.

## Conflicts of Interest

The authors declare no conflicts of interest.

## Supporting information


**Table S1:** Brazilian Portuguese Final Version of the Primary Care Network Assessment Scale for Patients in Palliative Care (ARCP).

## Data Availability

The data that support the findings of this study are available on request from the corresponding author. The data are not publicly available due to privacy or ethics restrictions.
