# Supplementary material for: Content Validation of Primary Care Network Assessment Scale for Palliative Care Patients (ARCP) and Clinical Utility in Patients With Advanced Cancer
Source: Scand J Caring Sci. 2026 Aug 6;40(3):e70303. doi: 10.1111/scs.70303 (PMC13448353; doi:10.1111/scs.70303)
Supplement: Supplementary file 1 [file SCS-40-0-s001.docx]

Supplementary Table 1: Brazilian Portuguese Final Version of the Primary Care Network Assessment Scale for Patients in Palliative Care (ARCP Scale).

| **Classificação** | **Tipo de rede** | **Quanto a existência de vínculos familiares e afetivos** | **Quanto à existência de cuidadores** | **Quanto ao quantitativo de cuidadores** | **Quanto a qualidade do cuidado ofertado** | **Conceito** |
| --- | --- | --- | --- | --- | --- | --- |
| 1 | Rede   social | Inexistente | *Não se aplica* | *Não se aplica* | *Não se aplica* | Quando não foi identificado nenhum suporte familiar ou de pessoas com vínculos afetivos para a oferta de cuidados ao paciente. |
| 2 | Rede social | Existente | Não participativa | *Não se aplica* | *Não se aplica* | Quando identificados familiares e/ou indivíduos com vínculos afetivos com o paciente, mas que não se organizam em rede para a oferta de cuidados. |
| 3 | Rede primária de cuidados | Existente | Participativa | Restrita | Parcial | Quando identificado apenas um cuidador que não abarca todas as demandas apresentadas pelo usuário. |
| 4 | Rede primária de cuidados | Existente | Participativa | Moderada | Parcial | Rede que envolve a ação de duas pessoas cuja ação não abarca todas as demandas apresentadas pelo usuário. |
| 5 | Rede primária de cuidados | Existente | Participativa | Ampla | Parcial | Rede que envolve a ação de três ou mais pessoas, e que não abarca todas as demandas apresentadas pelo usuário. |
| 6 | Rede primária de cuidados | Existente | Participativa | Restrita | Plena | Quando identificado apenas um cuidador cuja forma de ação abrange a integralidade do cuidado. |
| 7 | Rede primária de cuidados | Existente | Participativa | Moderada | Plena | Rede que envolve a ação de duas pessoas cuja ação abrange a integralidade do cuidado. |
| 8 | Rede primária de cuidados | Existente | Participativa | Ampla | Plena | Rede que envolve a ação de 3 ou mais pessoas cuja ação abrange a integralidade do cuidado. |
